# Dietary Fructose: A Literature Review of Current Evidence and Implications on Metabolic Health

**DOI:** 10.7759/cureus.74143

**Published:** 2024-11-21

**Authors:** Vishal Agarwal, Sambit Das, Nitin Kapoor, Binod Prusty, Bijay Das

**Affiliations:** 1 Endocrinology, Diabetes and Metabolism, Kalinga Institute of Medical Sciences, Bhubaneswar, IND; 2 Endocrinology, Diabetes and Metabolism, Christian Medical College and Hospital, Vellore, IND

**Keywords:** diabetes, fatty liver, fructose, hypertension, hyperuricemia, metabolic syndrome

## Abstract

With the increasing intake of dietary fructose, primarily from sucrose and sweetened beverages, metabolic illnesses such as type 2 diabetes mellitus, hypertension, fatty liver disease, dyslipidemia, and hyperuricemia have become more prevalent worldwide, and there is also growing concern about the development of malignancies. These negative health impacts have been validated in various meta-analyses and randomized controlled trials. In contrast, the naturally occurring fructose found in fruits and vegetables contains only a minimal amount of fructose and, when consumed in moderation, may be a healthier choice. This review focuses on the biology of fructose, including its dietary sources, the physiology of its metabolism, and the pathological basis of various disorders related to high dietary fructose intake.

## Introduction and background

The word “sugar” is derived from shakara, which means gravel in Sanskrit. Sugarcane cultivation in Bharat (India) dates back to 5,000 BC. Upon their arrival in Bharat in 327 BC, Alexander the Great’s soldiers were astonished to discover an alternative food sweetener to honey, describing the crop as a “reed that gives honey without bees.” This is how sugar was introduced to the world [1].

Until the 18th century, the human diet included only small amounts of sugars in the form of fructose and sucrose, which were naturally present in fruits, honey, and vegetables. With the introduction of newer technologies to the food industry, rapid industrialization facilitated the extraction of starch from corn. The subsequent hydrolysis of starch into glucose and isomerization of glucose into fructose led to a significant increase in sugar consumption in the 1960s [2]. This process resulted in the production of high-fructose corn syrup (HFCS), a corn-derived sweetener that was relatively cheaper, sweeter, and more soluble than sucrose, with the ability to remain in solution without crystallizing, as can sucrose under certain conditions [3]. Moreover, HFCS’s liquid nature made it easier to transport, which contributed to its widespread use in soft drink formulations. Due to its acidic nature, long shelf life, and low cost, HFCS also became a major component of the baking industry. HFCS provides little to no nutritional value beyond the calories from sugar. Given the increased intake of fructose over the past few decades, along with the rising rates of diabetes and metabolic syndrome, the impact of a high-fructose diet on individual health has garnered increased attention [4]. This paper describes the structure, biology, and clinical impact of fructose on various metabolic disorders.

## Review

Structure

**Figure 1:**
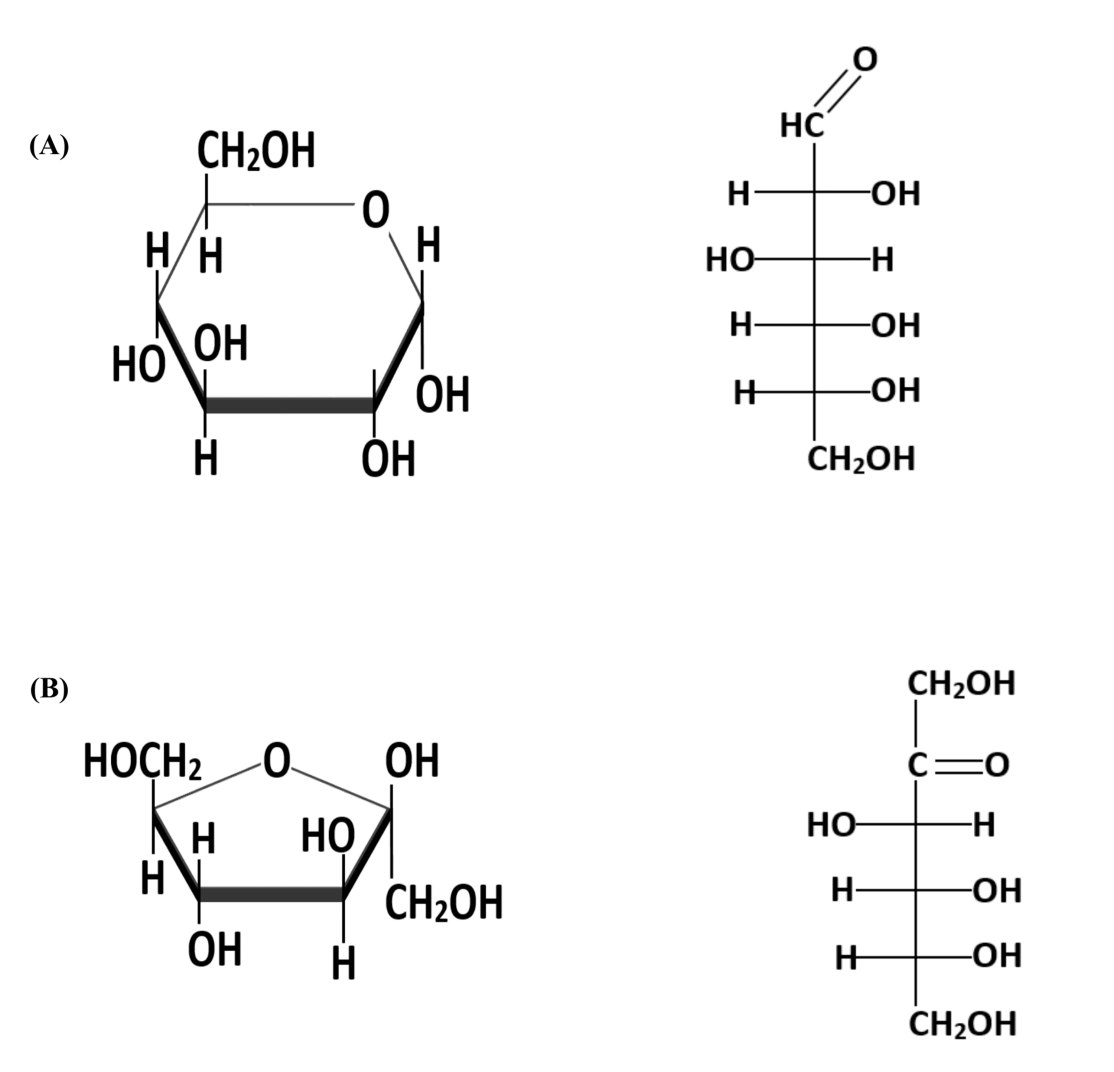
Structures of glucose (A) and fructose (B). Image credits: Sambit Das; Vishal Agarwal.

Fructose is a six-carbon atom, cyclic monosaccharide. Unlike the L-form, the D-form of fructose is commonly found in nature. While an isomer of glucose, which contains an aldehyde group at carbon 1, fructose is a ketose-hexose with a ketone group at carbon 2. Figure 1 shows the structures of glucose and fructose.

Dietary sources of fructose

Dietary fructose can be obtained as a monosaccharide from natural sources such as fruits, vegetables, and honey. Conversely, table sugar, which is available in the form of sucrose, is a disaccharide composed of 50% glucose and 50% fructose. HFCS is a major source of dietary fructose in the modern diet. The refining process of corn starch produces HFCS. Commercially, HFCS is commonly available in two forms, one containing 42% fructose and the other containing 55% fructose. Additionally, HFCS-90, which is a highly concentrated form of HFCS containing 90% fructose and 10% glucose, is sometimes used commercially [5]. Owing to its stability in acidic foods and low price, HFCS has become an ideal alternative sweetener to sucrose in the food and beverage industry.

Biology of fructose metabolism

Fructose differs from glucose in terms of gut absorption, first-pass metabolism in the liver, and subsequent metabolic pathways. In addition, its metabolism is not insulin dependent. Ingested dietary fructose, mainly in the form of sucrose, is hydrolyzed by the enzyme sucrase, which is present on the brush border cells of the intestinal epithelium. Sucrase cleaves sucrose into its component monosaccharides: glucose and fructose. Their entry into the intestinal epithelial cells is mediated by the gut transporters sodium-glucose transporter 1 (SGLT-1) and glucose transporter (GLUT) 5, respectively [6]. Due to the limited capacity of intestinal fructose absorption, this monosaccharide is absorbed more slowly than glucose. When ingested in large quantities, fructose remains in the intestinal lumen long enough to predispose to bacterial fermentation. This causes the formation of short-chain fatty acids (acetate, propionate, and butyrate), gases (hydrogen, methane, and carbon dioxide), and gastrointestinal discomfort. Fructose absorption is enhanced by the presence of glucose in the diet, the mechanism of which is not clearly known but is probably due to the effect of glucose on fructose transporters. Naturally occurring fructose in fruits and vegetables is always present alongside glucose [7].

**Figure 2:**
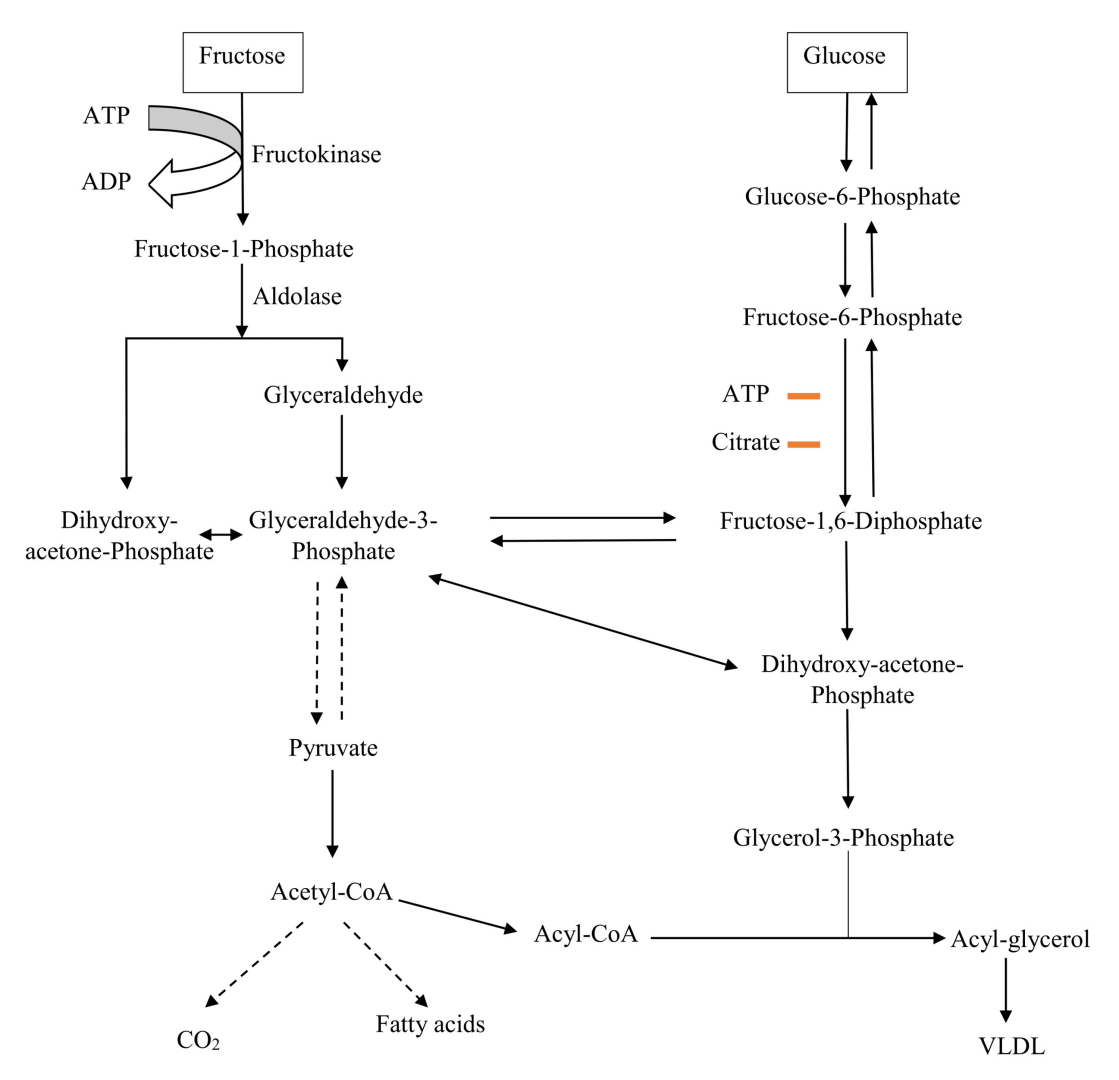
Fructose metabolism in the liver. ATP: adenosine triphosphate, ADP: adenosine diphosphate, VLDL: very low-density lipoprotein. Image credits: Sambit Das; Vishal Agarwal.

After its absorption in the gut, fructose enters the portal circulation and reaches the liver, where nearly 70% of it is metabolized by hepatocytes (Figure 2) on the first pass, compared to only 15%-30% of ingested glucose. Fructose bypasses the rate-limiting step of glycolysis (i.e., phosphofructokinase), which is tightly regulated by the cell’s energy status, and enters the later stages of the glycolytic pathway. GLUT 2 mediates the transport of fructose into hepatocytes, where it is immediately acted upon by the enzyme ketohexokinase-C, which converts fructose into fructose-1-phosphate (F-1-P). Unlike phosphofructokinase in glycolysis, this enzyme is not inhibited by adenosine triphosphate (ATP). This enzymatic activity remains unregulated, even with high ATP levels in the liver. Subsequently, the enzyme aldolase B cleaves F-1-P into dihydroxyacetone phosphate and glyceraldehyde. The latter is then converted into glycolytic intermediate glyceraldehyde 3-phosphate by the enzyme triose kinase, using ATP as the phosphate donor. Fructose then follows the glycolytic pathway, with its carbons further metabolized to form pyruvate, which feeds into the tricarboxylic acid cycle, producing citrate and providing acetyl-CoA for de novo fatty acid synthesis (lipogenesis) via the ATP citrate lyase enzyme [8].

Fructose and obesity

Since the introduction of HFCS as a sweetening agent in foods and beverages in the 1960s, the obesity epidemic has grown. Several intervention trials have shown a causal relationship between obesity and a high-fructose diet. A study conducted by Cox et al. [9] demonstrated that consuming high amounts of fructose (25% of energy requirement) for 10 weeks decreases resting energy expenditure and leads to obesity. Another study showed a reduction in body mass index in overweight and obese children after a decrease in oral fructose intake over three months [10].

Additionally, animal studies have shown that fructose intake is associated with higher leptin concentrations [11]. Adipose tissues synthesize the hormone leptin, which interacts with hypothalamic centers to induce satiety and energy expenditure. High fructose intake is linked to leptin resistance, leading to increased food intake [12]. Central pathways that are modulated by fructose and affect satiety regulators include reduced mRNA expression of peptide YY, neuropeptide Y, and proopiomelanocortin, as well as the increased mRNA expression of cannabinoid 1 [13]. These changes can contribute to increased calorie intake and obesity. Another mechanism by which fructose stimulates hunger is by depleting ATP in the liver through the inhibition of fatty acid metabolism, a characteristic feature of fructose metabolism. Furthermore, it is noteworthy that ingesting sugar has a pleasurable effect by stimulating dopaminergic neurons in the nucleus accumbens and midbrain. In short, obesity caused by fructose intake may result from the combined effect of leptin resistance, an imbalance in central satiety regulators, the stimulation of hunger via ATP depletion in the liver, and the pleasurable effect of sugar through dopaminergic pathways [13].

Fructose and gut dysbiosis

The Firmicutes-to-Bacteroidetes ratio is altered in rodents fed with a fructose-rich diet, leading to changes in the gut microbiota [14]. Higher levels of fructophilic lactic acid bacteria have been observed in rodents on a fructose-rich diet. Gut dysbiosis, characterized by an increased Firmicutes-to-Bacteroidetes ratio, has been linked to the development of obesity in humans [15].

Fructose and inflammation

High-fructose diets induce a pro-inflammatory state in the body. Studies conducted in both humans and rodents have shown that the expression of pro-inflammatory cytokines, such as tumor necrosis factor-α and interferon γ, is markedly increased with excessive dietary fructose intake. Additionally, levels of inflammatory markers, such as C-reactive protein, are elevated [16]. Moreover, high-fructose diets increase systemic oxidative stress by promoting the release of reactive oxygen species [17].

Fructose and insulin resistance

The chronic consumption of fructose leads to skeletal muscle fat accumulation, inducing peripheral insulin resistance and de novo lipogenesis (DNL), along with increased triglyceride content and fat accumulation in skeletal muscles and pancreatic β-cells. These changes contribute to an increase in peripheral insulin resistance and lipotoxicity of β-cells, thereby increasing the risk of diabetes [18]. Chronic fructose intake also reduces adiponectin, a hormone produced by adipocytes, which may contribute to peripheral insulin resistance. Adiponectin is essential for the expression of molecules in skeletal muscle cells that facilitate fatty acid transport and reduce insulin resistance [19,20].

Studies investigating the direct effect of fructose on insulin resistance or evaluating insulin signaling pathways are scarce. However, a few studies have suggested that high dietary fructose intake may be linked to defects in insulin receptor substrates 1 and 2 and in the phosphorylation or activation of phosphatidylinositol-3 kinase, leading to insulin resistance [21]. Insulin resistance, along with obesity, is a precursor to type 2 diabetes mellitus. Furthermore, meta-analyses have clearly shown that individuals with a high intake of sugar-sweetened beverages (SSBs) containing fructose have a higher risk of developing obesity, metabolic syndrome, gout, and type 2 diabetes mellitus [22].

Fructose and metabolic dysfunction-associated steatotic liver disease

Chronic fructose intake is associated with metabolic dysfunction-associated steatotic liver disease (MASLD), which occurs independently of weight gain. Clinical trials have shown that dietary fructose intake is two to three times higher in children and adults with MASLD compared to control individuals without fatty liver disease [23].

Chronic fructose intake stimulates DNL and inhibits fatty acid oxidation, leading to the deposition of triglycerides and the development of hepatic steatosis [24]. Additionally, high dietary fructose intake alters the intestinal microbial flora, promoting the translocation of gut microbes and increasing lipopolysaccharide concentrations in systemic circulation [25]. Owing to elevated endotoxemia, the secretion of tumor necrosis factor is increased, which activates the transcription factor SREBP1c, one of the key regulators of DNL, inducing hepatic steatosis. The gut microbiota also converts fructose into acetate, independently of the mucolytic metabolic pathway, supplying lipogenic acetyl-CoA to the liver [26]. Fructose metabolism in the liver also generates more reactive oxygen species compared to glucose, which can cause further hepatic damage and fibrosis [27].

In a study conducted by Silbernagel et al. [28], 20 healthy individuals with an elevated mean body mass index of 25.9 kg/m^2^ and normal hepatic fat content at baseline (1.5%) were given a high-fructose diet for four weeks. At the end of this period, no change in hepatic fat content or insulin resistance was observed, as assessed by magnetic resonance imaging. A small sample size limited the validity of this study. Another study involving a short-term (nine days) fructose-restricted diet showed a decrease in liver fat content and improved insulin resistance in children with obesity [29]. The most conclusive evidence that fructose induces hepatic steatosis comes from a six-month randomized clinical trial comparing SSBs to noncaloric drinks and milk. Magnetic resonance imaging was used to assess relative changes in hepatic fat content, which was significantly increased in the fructose group [30].

Fructose increases fatty acid synthesis and triggers de novo hepatic lipogenesis. Equally, through its metabolites, it prevents β-oxidation-mediated lipid breakdown, which leads to the formation of visceral fat. These processes cause chronic inflammatory damage and persistent fibrosis in hepatocytes, enabling MASLD to progress to metabolic dysfunction-associated steatohepatitis and, ultimately, liver cancer [31].

Fructose and hypertension

In the past few decades, the rising incidence of hypertension has paralleled the increased intake of fructose [32]. HFCS present in beverages is a major source of dietary fructose, and many researchers have identified a causal relationship between fructose intake and hypertension. Studies have shown that high dietary fructose intake is a major risk factor for the development of hypertension [33]. Participants in the study conducted by Endo et al. experienced a prolonged rise in mean blood pressure for two hours after consuming 50 g of oral fructose. This increase was most likely caused by an elevated cardiac output that was not offset by peripheral vasodilation [34]. In animal studies, rats fed a high-fructose diet for 18 weeks also exhibited a significant increase in systolic and diastolic blood pressure, along with elevated angiotensin II levels [35]. However, the effect of fructose consumption on blood pressure has shown variable results across different studies. In one study, seven young, nonsmoking White male volunteers participated in a four-week prospective trial, and after receiving 1.5 g/kg/day of fructose supplementation, no change in mean blood pressure was observed [36]. Similarly, a trial involving 85 healthy participants who consumed beverages with varying amounts of HFCS (10%, 17.5%, and 25% of the energy requirement percentage) for 15 days showed no significant increase in blood pressure [37]. On the other hand, a study where participants received 200 g of fructose daily for 15 days revealed significant effects on blood pressure, serum uric acid, and lipid markers. After this period of fructose supplementation, systolic and diastolic blood pressure increased by 7 ± 2 mmHg and 5 ± 2 mmHg, respectively. These increases were mitigated by allopurinol medication, indicating that uric acid may play a role in mediating the effect of fructose on blood pressure [38]. Additionally, some studies have suggested the potential role of GLUT 5 in the pathophysiology of hypertension. In a study conducted by Barone et al., GLUT 5-/- mice developed malabsorption of fructose along with weight loss and hypotension. In contrast, the GLUT 5+/+ mice developed hypertension after 14 weeks on a high-fructose diet [39]. Several theories have been postulated to explain how high dietary fructose intake leads to hypertension, including endothelial dysfunction induced by hyperuricemia, increased sympathetic tone, and activation of the renin-angiotensin-aldosterone system, which may contribute to elevated blood pressure. Thus, further studies in this area are needed to better understand the underlying mechanisms of fructose-induced hypertension.

Fructose and cardiomyopathy

Animal studies have highlighted the occurrence of systolic dysfunction, apoptotic cardiomyopathy, and ventricular wall thinning in rats fed with an excessive fructose diet [40]. Several theories have been postulated to explain these effects, including the development of metabolic syndrome induced by a high-fructose diet, hyperuricemia, and overexpression of monocyte chemoattractant protein-1 (MCP-1) on cardiac myocytes, which leads to cardiac inflammation. Furthermore, increased susceptibility to myocardial ischemia has been observed in rats fed with a high-fructose diet [41]. 

Fructose and hyperuricemia

The incidence of hyperuricemia has paralleled the rising trend in HFCS intake over the past few decades and is often characterized by the deposition of urate crystals in synovial fluid and other tissues [42]. As mentioned earlier in the “Biology of fructose metabolism” section, fructose is phosphorylated by ketohexokinase-C, which converts fructose into F-1-P, and unlike phosphofructokinase in glycolysis, this enzyme is not inhibited by ATP, leading to local ATP depletion and increased levels of AMP, which is a precursor to uric acid formation. The prevalence of hyperuricemia is estimated at 3.9% in the United States, with a 0.12%-4% incidence in the Asia-Pacific region [43]. Estimates of the overall increase in gout prevalence have been made, considering the increased consumption of fructose-laden carbonated drinks [44]. Several studies have helped clarify the relationship between high dietary fructose intake and hyperuricemia, though the findings show some disagreement. An augmented risk of gout has been associated with the intake of SSBs and fructose, as demonstrated in cohort studies [45,46]. Increased postprandial uric acid levels were observed in healthy individuals in a randomized controlled crossover trial after consuming HFCS compared with sucrose-sweetened soft drinks [47].

Fructose and malignancy risk

Recent research has raised concerns about the potential link between high fructose intake and an increased risk of certain types of cancer, especially those affecting the gastrointestinal system. Researchers observed an increase in the size and grade of colorectal tumors in mice with mutant adenomatous polyposis coli genes. The expression of GLUT 5 was increased in the intestinal tumors of these mice and also in human colonic cancers compared to normal colonic epithelial cells [48]. A meta-analysis of 13 studies found a positive association between high dietary fructose intake and an increased risk of pancreatic cancer, demonstrating a link between carbohydrate intake and pancreatic cancers. A plausible explanation for this link is that the liver, being the primary site of fructose metabolism, may generate reactive metabolites and promote the buildup of uric acid. The development of cancer has been linked to oxidative stress and inflammation, both of which are induced by high uric acid levels. Moreover, fructose-induced activation of specific signaling pathways, including those involved in cellular growth (such as the mTOR pathway) and insulin resistance, may promote tumor development [49,50].

## Conclusions

Fructose is a naturally occurring monosaccharide with a sweet taste. Consumption of dietary fructose in moderate amounts, ranging from 25 to 40 g/day, is considered safe. However, excessive intake of fructose can lead to various negative health outcomes, such as DNL, metabolic syndrome, type 2 diabetes, hypertension, gout, and certain malignancies. High dietary fructose consumption also increases the risk of developing insulin resistance and obesity. Given its potential negative health impacts, consuming large amounts of fructose may lead to adverse health consequences. Unlike SSBs containing HFCS, fruits and vegetables naturally contain only moderate amounts of fructose and may be a healthier option when consumed in moderation.
